# The Strain of War on the Nervous System

**Published:** 1917-08-25

**Authors:** 


### THE STRAIN OF WAR ON THE NERVOUS SYSTEM.

### Efficiency in Politicians and Soldiers.

The mental strain inflicted by the war is a very real and very widespread influence. In reference to individuals it falls, of course, with markedly unequal incidence, and some are able to sustain a burden under which others succumb. Further, the strain is an increasing quantity. Here, as in other experiences, there is at the outset abundant vigour and enthusiasm. The ideal or aim is conspicuous, and it obliterates from the view the wearisome and toilsome way which must be travelled in order to compass it. The heart is young, the mind fresh, the body alert. All the physiological machinery, psychical as well as physical, is at the height of its condition. But as time advances the situation changes. The elevated motive becomes confused by side issues. Controversies and differences emerge. The mental stimulus becomes less penetrating, and it falls on an organisation less capable of full and prompt response. Losses and disasters and personal sorrows multiply, and give rise to uneasy questionings. Thus, in the very nature of things, the nervous apparatus undergoes a kind of progressive depreciation, and weariness and staleness arrive. The way is found to be long and weary, and even courage, resolution, and fortitude feel the hardness of it.

### The Scientific Basis of a Popular Doctrine.

Such are the consequences of every prolonged labour and effort. To look at the stars is easy enough at the beginning, but the dust and mire of the road assert themselves in increasing measure, and the downward glance becomes a more and more frequent experience. A history of this order is being plainly written by the war, and the effects can be seen both in the community generally and in the individual. Statesmen have to take note of such a state of matters, and thus they recognise the need for national encouragement as an antidote to national weariness. To keep the heart of the nation high and to steel its determination is part of the patriotic duty of all those who speak to the public ear, and this demand arises out of the circumstances which are an inevitable part of prolonged strain and sustained effort. The processes both in one direction and the other have a physiological basis, and could we unlock all the secrets of the nervous system we should doubtless find the mystery of the mechanism and the conditions of its working. Fatigue is a familiar theme to the modern student of physiology, and recently its bearing on muscular effort and mechanical work has been systematically examined. The result has been to place on a scientific basis the truth contained in the popular doctrine that "all work and no play makes Jack a dull boy." It has done more than this. It has shown that the sharp edge of effort begins to fail first in the nervous system, and this, be it remembered, is found to be true, not merely of mental work but also of work in which the most conspicuous element is muscular strain. Such a conclusion would hardly have been expected in advance, but it is established by the most rigorous scientific methods. It is this fatigue of the nervous system which, as it gradually becomes established, lessens both the quantity and quality in the output of the work undertaken. Hence the physiological need for the interruption of labour by rest, if a high standard of accomplishment is to be sustained. Still more, the investigations which have established these propositions as secure beyond challenge have also indicated that their economic importance is a considerable one. In other words, if work and stress are continued beyond the point at which fatigue invades competence, it is found that the total output over a given period is less than when fatigue is met by properly measured intervals of rest. Economy and efficiency, therefore, not less than popular impulse and physiological measurements, call for the mutual adjustment of work and rest if the bodily machinery is to be used to the best advantage, and if also the products of its labour are to reach the highest level both in sum and character.

### Resting Periods for All.

Apply these truths to the subject of war weariness and their significance can hardly be missed. Unhappily the war continues, and so far as the omens can be judged is likely to continue. With it the consequential strain increases, as one may say, in geometrical ratio. Inevitably, therefore, there is danger of deterioration in the efficiency of those who have to conduct the war. It will be remembered that when Lord Kitchener, at the very outset, contemplated a three years' struggle he recognised that should the effort have to be more prolonged there would probably be need for new men to take up the burden. For himself he accepted a quasi-political office with the plain indication that at the end of the three years' limit he would have to reconsider his position; and what was true for himself he recognised would probably be true for others. The date Lord Kitchener suggested has now been overpassed, and it is appropriate to consider the truth which is implicit in his doctrine. Some men have been under great and continuous strain almost throughout the whole period. It is inevitable that in the circumstances their work should suffer as well as themselves. We cannot, of course, abruptly order a new set of national leaders or an army of soldiers, or arrange a cut-and-dried series of "shifts" as in a factory or coal-mine. But we can endeavour to secure a plan by which our politicians and soldiers shall do their work under the protection of scientific law. In other words, it is a national interest and an interest of efficiency that there shall be resting periods for all who contribute in their several spheres to the sustained effort which the war imposes.

To urge this upon our politicians in reference to themselves may be defended as a medical duty No very penetrating eye is necessary to see that neglect of it is having unfortunate consequences, and these apply to Cabinet Ministers as well as to those who labour with their hands, or perhaps more forcibly to the former than to the latter. Even in the crisis which threatened last week it is not difficult to suspect that overstrained nervous systems have played a not inconspicuous part. Misconceptions, misunderstandings, forgetfulness, and blurred mental vision are the inevitable expression of strained and tired brains, and in large national affairs they may lead to enormous disaster. We cannot "run" the country with successive relays of leaders, for continuity of policy and sustained action involve identity of *personnel*. But we can advise our leaders that, even as other men, they are under the sway of physiological law, and that if their work is to be of the first value, whether in kind or degree, they must conform to scientific truth. It was the fashion in certain quarters not very long ago to sneer at the week-end habit adopted by some of our leaders, and to set as a contrast to it the politicians who never failed at their desks, and who for seven days in each week, not to speak of the hours of the night, held themselves unbent over their labours. The doctrine can only be described as an expression of ignorance. What we want in these supreme offices above all else is efficiency, and efficiency by the method suggested is frankly impossible. Sooner or later the adoption of such advice will mean a hesitating judgment and an obscured outlook, nerves on edge and an explosive temper. If the country is to be saved from the disasters which these things imply our leaders must recognise what science teaches us are the necessary adjustments of work and rest. They, like their fellows, if they are to produce of their best, must bow to the imperious demands of physiological law. What has been demonstrated as true by rule and measure in the case of manual labourers must be accepted as not less applicable to those subjected to still higher stresses. Whatever the work to which it is put, the human nervous system remains fundamentally the same, and it requires appropriate conditions if its full measure of efficiency is to be maintained.

### The Case of the Soldiers.

If all this is true of the politicians and of the manual workers, it is not less true of the soldiers, and, indeed, it applies with special force to these. Here is the element not only of physical stress, but also of emotional strain and personal danger. In some ranks the one of these is predominant, in some others prevail. But in all, the nervous system inevitably suffers. The long story of the war inflicts its penalty, and efforts must be made to meet it. There is importance in the truth that the expression of this strain may take on such subtle forms as have been alluded to in the earlier part of this article. Not some open and gross breakdown, but a failure of tone and temper and judgment may be the warning voice. Neurasthenia is a wide term, and is one not easily defined. But it does express a real condition in which the nervous apparatus declines to react in a normal fashion both to the external world and to the sufferer's will. Not welcome in any circumstances, it is particularly obnoxious to the man of active life, and to the military mind it conveys almost a reproach. Thus the soldier is the last person to acknowledge it, so long as any measure of resistance is possible, for the public opinion of his world is hardly ready to admit its validity. In these circumstances there is necessarily included an element of danger, not only to the individual but also to the Service he directs.

### Methodical Adjustments Called for.

In an engaging article in the *British Medical Journal* of the 11th inst. this point of view has been forcibly and admirably presented. The writer surveys the position from a standpoint not less scientific than it is sympathetic, and proposes measures to meet it. Men in responsible command are reluctant, for reasons already explained, to recognise that if their usefulness is to be continued they must for a time give up active duties. The truth, however, remains, and it commands the situation. From this there arrives the opportunity for medical intervention. The duty of the Medical Service is to protect and preserve, not only the physical health of their comrades but also their mental health. They must prescribe the conditions which promote both the one and the other. The latter of these two aims is just now specially prominent, and it is to be attained by the appropriate measures which science demands. The time has fully come when by a routine procedure our fighting men, and especially those burdened with large responsibilities, should escape for a time and at regular intervals from the strain which their duties inflict upon them. In this way can be secured what is essential if their work is to be efficient, and it can be secured with a minimum of inconvenience. So long as release only comes through personal application and by the agency of individual medical officers and examining boards, the conscientious and sensitive officer will shrink from seeking it. He will deny that he is an invalid needing sick-leave, and will hold on to his duty to the last gasp. From this invidious position it is necessary to save him, and the salvation can only be secured by the method here indicated. The higher medical authorities in the Army have not hesitated to press on the military Commands the measures necessary to the physical well-being of our armies, and they have their justification in openly displayed facts. We urge them to be equally emphatic in reference to the more subtle dangers which are here sketched in outline. At a pinch, chances must be accepted and prudence set at defiance. But the war is less an emergency than a sustained and telling strain. It demands methodical adjustments in all directions, and not the least important of these is an arrangement by which our soldiers shall be brought under the conditions which are essential to efficiency. The soldiers do not know these conditions. But the doctors know them, and it is therefore the duty of the medical profession to see that they are put into operation.

